# A Comprehensive Research Review of Herbal Textual Research, Phytochemistry, Pharmacology, Traditional Uses, Clinical Application, Safety Evaluation, and Quality Control of *Trollius chinensis* Bunge

**DOI:** 10.3390/ph17060800

**Published:** 2024-06-18

**Authors:** Keke Yang, Zhen Wang, Panpan Wang, Lai Wang, Yuanjie Li, Lianqing He, Xiubo Liu, Jiao Xu, Yijin Duan, Wei Ma

**Affiliations:** 1 Pharmacy of College, Heilongjiang University of Chinese Medicine, Harbin 150040, China; 18904544819@163.com (K.Y.); wz870220@126.com (Z.W.); 15134532248@163.com (P.W.); 19145689707@163.com (Y.L.); hhelianqing@126.com (L.H.); xujiao2007@sina.com (J.X.); 2 College of Jiamusi, Heilongjiang University of Chinese Medicine, Jiamusi 154007, China; 18246179827@163.com (L.W.); zyylxb@126.com (X.L.); d2240061468@126.com (Y.D.)

**Keywords:** *Trollius chinensis* Bunge, flavonoids, organic acids, alkaloids, pharmacodynamic activity, clinical application

## Abstract

*Trollius chinensis* Bunge (TCB) is a perennial plant of the Ranunculaceae family with medicinal and edible values. It is widely distributed and commonly used in various regions, including Asia, Europe, and North America. The main chemical components of TCB include alkaloids, flavonoids, phenolic acids, and volatile oil compounds. TCB is renowned for its anti-inflammatory, heat-clearing, detoxifying, and eyesight-improving properties. Its dried flowers are commonly used as a traditional Chinese medicine indicated for the treatment of upper respiratory tract infections, chronic tonsillitis, pharyngitis, influenza, and bronchitis. Modern pharmacology has demonstrated the anti-cancer, anti-inflammatory, antihypertensive, and antioxidant effects of TCB. This study presents a comprehensive overview of various aspects of TCB, including herbal textual research, botany, phytochemistry, pharmacology, traditional uses, clinical application, and quality control, aiming to provide new ideas on the scientific application of TCB as well as the integration of modern research with traditional medicinal uses.

## 1. Introduction

TCB is a traditional medicinal plant of the Ranunculaceae family, which can remove heat and toxins from the body [1,2]. Its medicinal value was first described in the book Supplements to Compendium of Materia Medica. TCB is predominantly distributed in the temperate and cold temperate regions of the northern hemisphere and has extensive application in northern China, Inner Mongolia, Russia, and numerous other locations [3]. TCB has a bitter flavor and is non-toxic. TCB is the main treatment for mouth sores, throat swelling, floating heat tooth Xuan, earache, eye pain, bright eyes, and solving the Arashi barrier [4]. In addition, TCB’s principal uses are for treating respiratory tract infections, namely, pharyngitis, tonsillitis, and bronchitis [5]. TCB has significant antibacterial, antimicrobial, antiviral, anti-tumor, antioxidant, and other activities [6]. In 2023, TCB was selected as one of the components in a drug for the prevention of severe acute respiratory syndrome-coronavirus-2. Upon decoction with water, the formulation was shown to clear heat, remove toxins, and disperse wind. Gundy et al. found that a TCB “soup” may have the potential to fight against new coronaviruses [7].

Modern pharmacology shows that TCB has antiviral, antioxidative, anti-tumor, anti-inflammatory, antibacterial, and other pharmacological effects [8]. Among them, the main pharmacological components are flavonoids, organic acids, and alkaloids [9]. TCB also contains a small amount of active ingredients, such as polysaccharides and volatile oils, which have antioxidant and other pharmacological effects. The trace components of TCB work in synergy with the main active ingredients, producing good pharmacological effects with few adverse effects, and have enormous research potential. The compatibility of TCB with other elements of traditional Chinese medicine could expand its medicinal value, such as the compatibility of Jinlian flower and dandelion for the treatment of acute and chronic tonsillitis [8] and the compatibility of Jinlian flower with chrysanthemum flower and raw licorice root for the treatment of acute otitis media, acute tympanitis, etc. [10].

This article provides a comprehensive review of the literature concerning the herbal research, botany, phytochemistry, pharmacology, and traditional uses of TCB. In addition, we review the clinical applications and quality control of TCB. The purpose is to provide a future reference for modern research on TCB.

## 2. Materials and Methods

The relevant literature comes from scientific databases, namely, CNKI (https://www.cnki.net/, accessed on 15 January 2024), WanFang Data (https://www.wanfangdata.com.cn/, accessed on 18 January 2024), SinoMed (http://www.sinomed.ac.cn/index.jsp, accessed on 21 January 2024), Embase (https://www.embase.com/, accessed on 23 January 2024), Google Scholar (https://scholar.google.cz/, accessed on 25 January 2024), and Baidu Scholar (https://xueshu.baidu.com/, accessed on 26 January 2024). In addition, this review includes the academic journals identified in SciFinder (https://www.cas.org/solutions/cas-scifinder-discovery-platform/, accessed on 27 January 2024), Web of Science (https://webofscience.clarivate.cn, accessed on 29 January 2024)), PubMed (https://pubmed.ncbi.nlm.nih.gov/, accessed on 30 January 2024), and other academic platforms, which were used to search for articles related to TCB. The related monobooks and the *Chinese Pharmacopeia* were also used as references. The research progress of TCB can be summarized from seven aspects: herbology, phytochemistry, pharmacology, traditional application, clinical application, toxicology, and quality control. *Trollius chinensis*, phenolic acids, flavonoids, pharmacological action, clinical application, and quality evaluation were used as keywords. We provide an overview of 360 relevant academic papers. This article brings together more than 140 studies on TCB.

### 2.1. Herbal Textual Research of TCB

**Figure 1:**
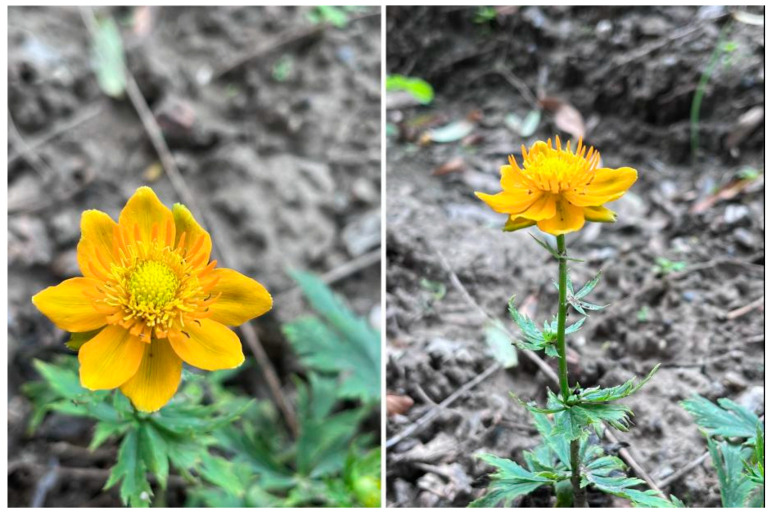
The plant of TCB.

TCB is a perennial herbaceous plant of the Ranunculaceae family, genus TCB (Figure 1). Its medicinal use was first described in Gleanings from the Compendium of Materia Medica by Zhao Xuemin in the Qing Dynasty in China. He said that TCB was “bitter in taste, cold in nature, and non-toxic” and could be used to “treat mouth sores, swollen throat, floating hot teeth, earache, and eye pain” [11]. Other books, including Guangqun medicinal compositions, Botanical names and facts, have been published discussing TCB [12]. The flowers have been described as “Golden plum grass”. Therefore, TCB’s name is related to its appearance, morphology, and growth environment. TCB was included in the 1977 edition of the Pharmacopoeia of the People’s Republic of China (hereinafter referred to as the Chinese Pharmacopoeia) but was not included in the subsequent editions. In 1998, TCB was included in the Drug Standard of the Ministry of Health of the People’s Republic of China–Mongolian Drugs Subsection. The 2020 edition of the Chinese Pharmacopoeia included six types of Chinese medicinal preparations based on TCB, which is also known as “tropaeolum”, “dry land lotus”, and “gold pimple” (Table 1).

**Figure 2:**
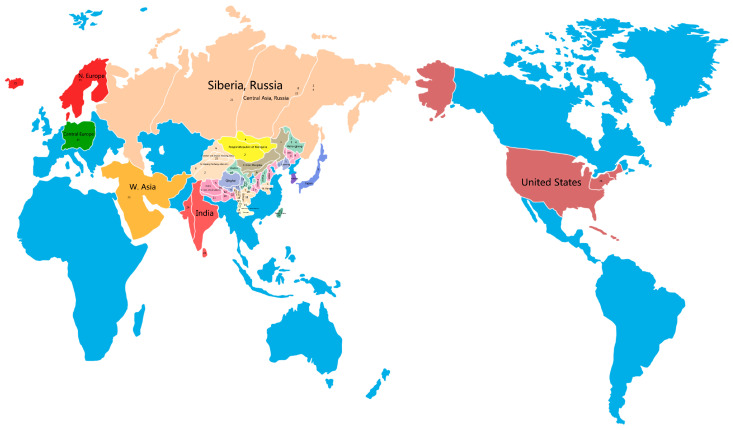
Distribution map of TCB.

According to the Dictionary of Traditional Chinese Medicine Resources, the China Plant Species Information Database, Plantwise, and other relevant websites, there are c. 32 species of *Trollius chinensis* species worldwide [24]. Of these, 26 species are distributed mainly in the temperate and cold–temperate mountainous areas in the Northern Hemisphere. Of these 26 species, approximately 16 species are from China, which are distributed mainly in the southwest, northwest, north, and northeast of China and Taiwan (Figure 2, Table 2) [24,25]. In Hebei Province, TCB was recorded in the early Qing Dynasty [26,27]. The Guang Qunfang Spectrum records TCB “Out of Shanxi Wutai Mountain” and the People’s Records states that dry TCB was “Produced in Shanxi” in China. The Hebei Manual of Traditional Chinese Medicine indicates that TCB is distributed in the “Northeast, Hebei, Shanxi and other provinces and regions” [28]. Chinese Herbal Medicine states that TCB is “Distributed in the northeast and Inner Mongolia, Hebei, Shanxi, and other places” [29]. Chinese Materia Medica reports that TCB is “Distributed in western Jilin, Liaoning, eastern Inner Mongolia, Hebei, Shanxi and northern Henan” [30].

One study shows that in the mountainous area of northern Hebei, the habitat of TCB was divided into the five following types: wetland meadows, forest clearings, undergrowth, ravines, and barren slopes. TCB had the highest distribution in wetland meadows and a lower distribution in ravines and barren slopes [31]. TCB and *Trollius asiaticus* L., *Trollius ledebourii* Rchb., *Trollius buddae* Schipcz., *Trollius farreri* Stapf., *Trollius ranunculoides*, and *T. asiaticus* L. were included in the Dictionary of Traditional Chinese Medicine. *T. farreri* Stapt, *Trollius ranunculoides* Hemsl., and *Trollius altaicus* C.A. Mey are also commonly used in traditional Chinese medicine formulations [32,33]. Due to the similarities in the morphology and efficacy of TCB and its congeners, there are different sources of TCB that are commercially available, and the standards in each locality are also different. The National Chinese Herbal Medicine Name List includes the medicinal plants TCB, *T. altaicus* C.A. Mey, broad-petaled TCB, *T. asiaticus* L., *T. buddae* Schipcz., *T. farreri* Stapf, *Trollius farreri* Stapf var. major. W.T. Wang, *Trollius japonicus* Mi, *Trollius japonicus* Changbaikai, *Trollius japonicus* Miq., short-valved goldenrod *T. ledebourii* Rchb., short-heavy-valved goldenrod *Trollius ledebourii* Rchb. f. plena (Kitag.) Y.C. Chu, long-valved goldenrod *Trollius macropetalus* Franch., *Trollius pumilus* D. Don, *T. ranunculoides* Hemsl., and *Trollius yunnanensis* (Franch.) Ulbr. [34].

### 2.2. Phytochemistry

**Figure 3:**
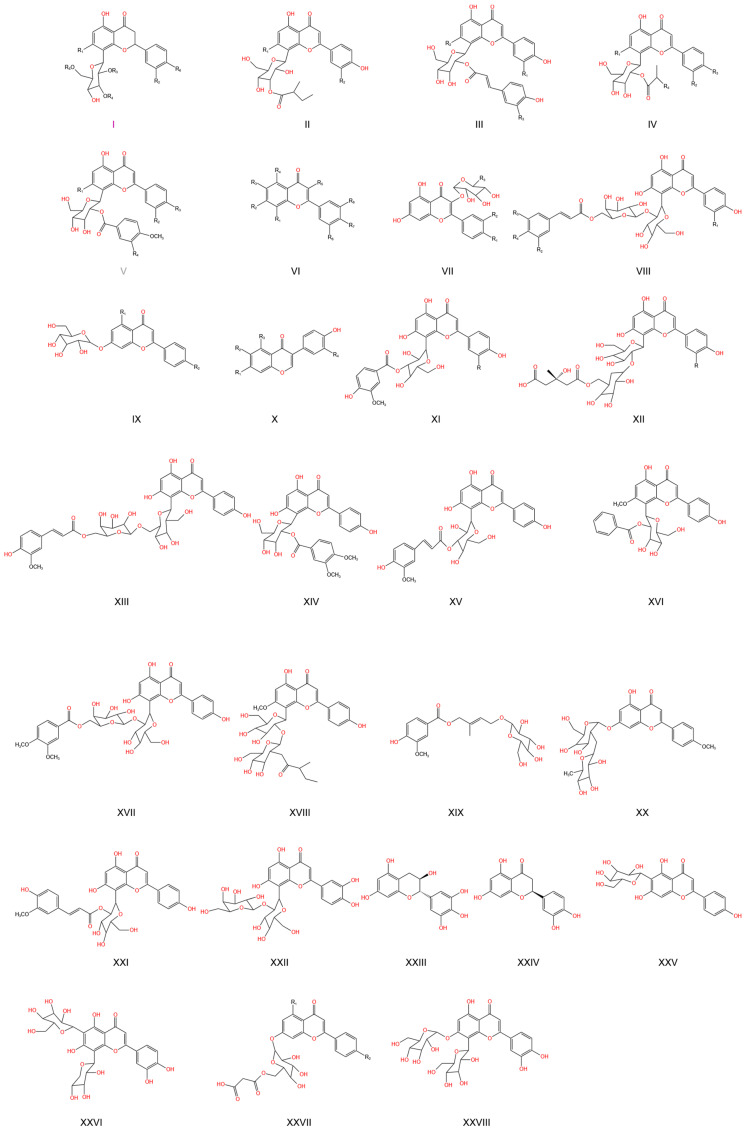
The matrix structure of flavonoid chemicals in TCB.

**Figure 4:**
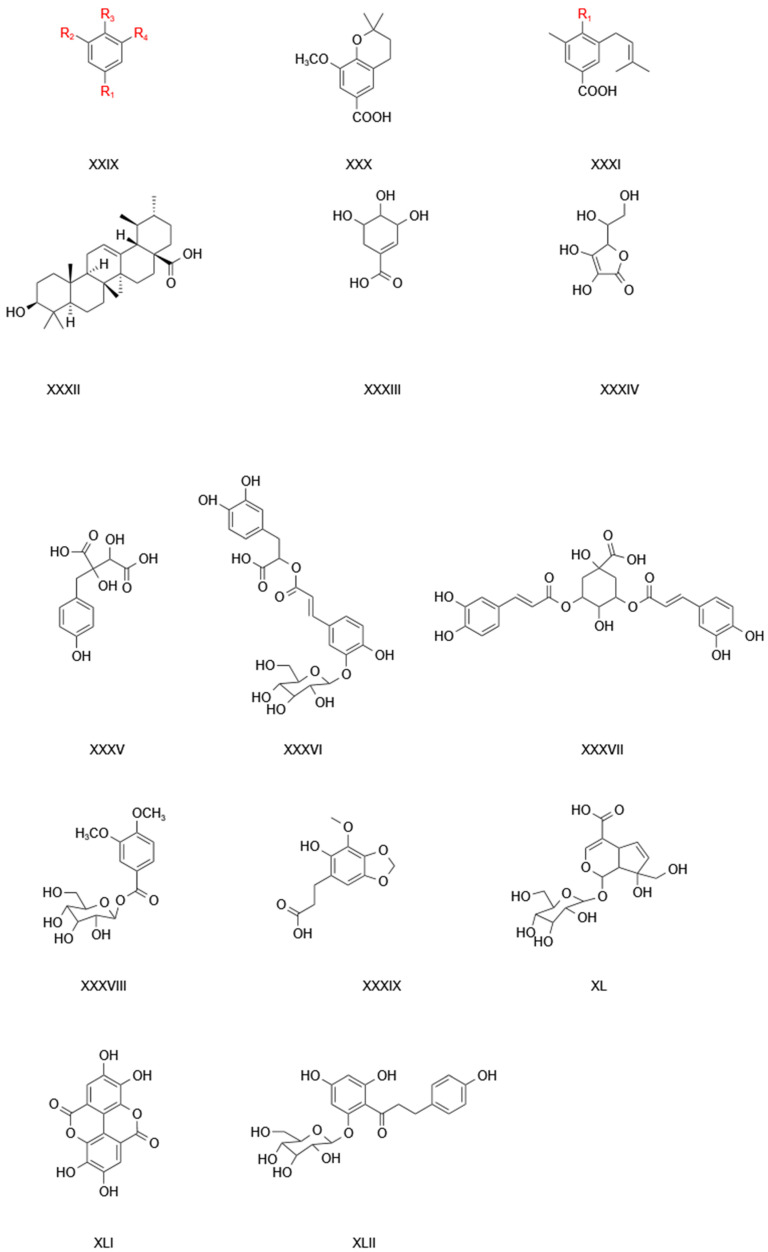
The structure of the parent nucleus of phenolic acid compounds in TCB.

**Figure 5:**
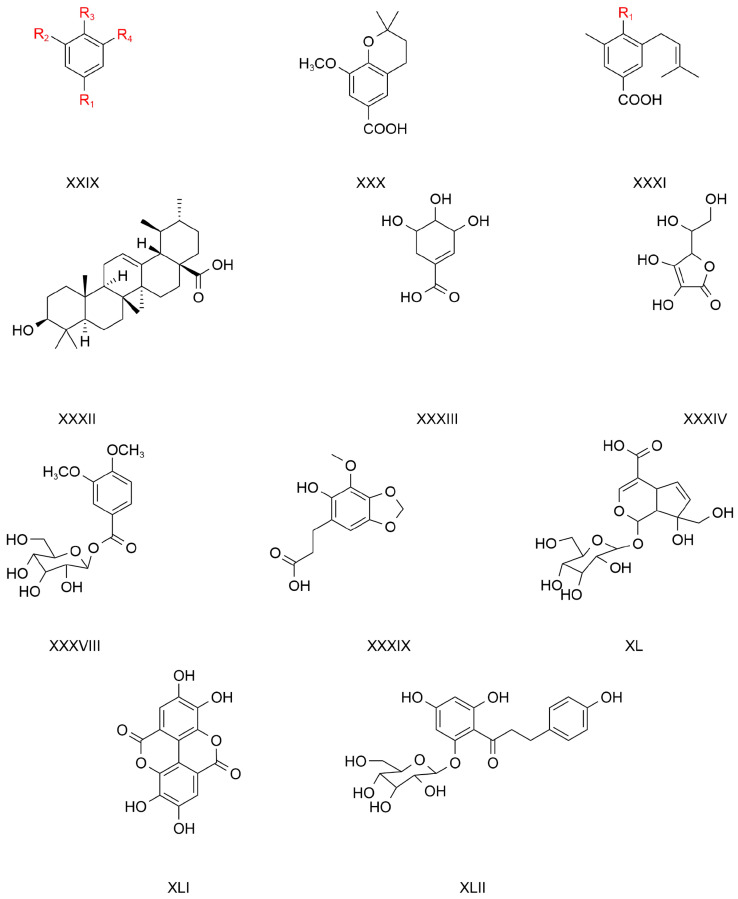
Structure of the parent nucleus of fatty acid analogs in TCB.

**Figure 6:**
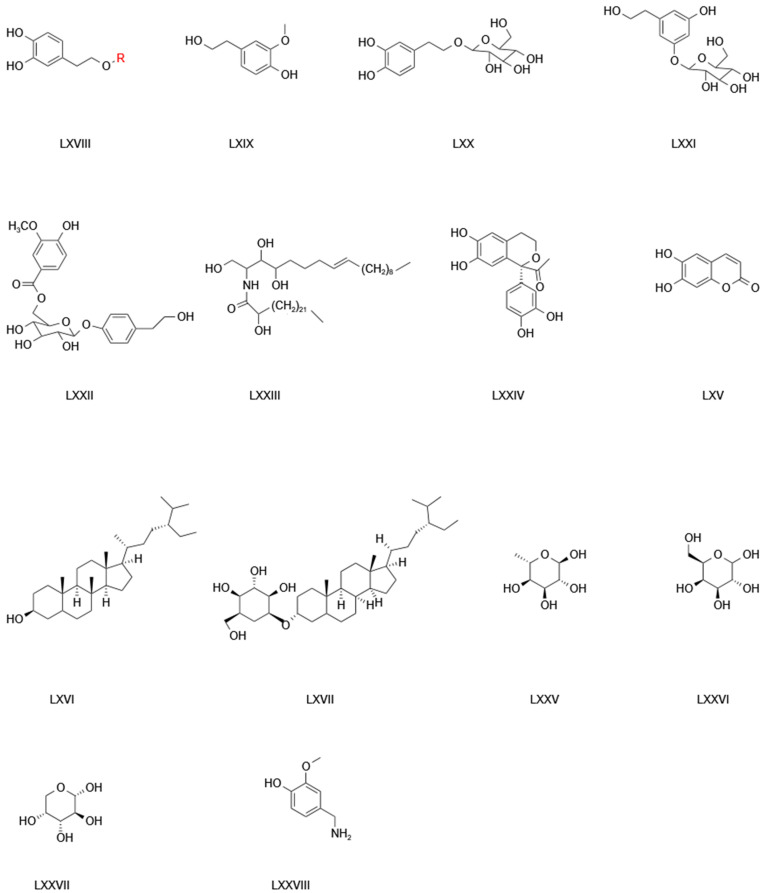
The structure of the parent nucleus of other compounds in TCB.

**Figure 7:**
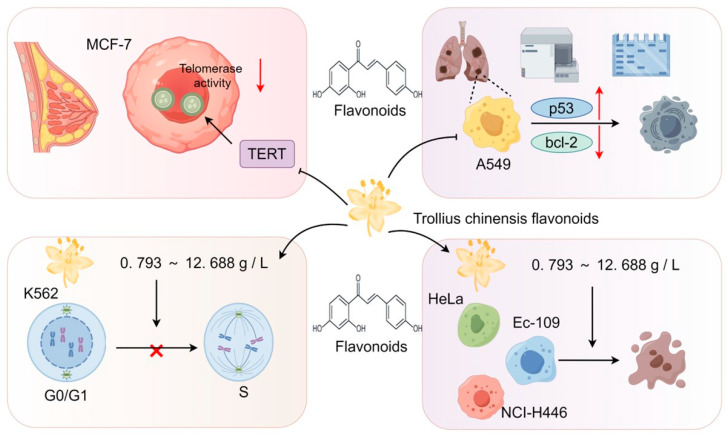
Anti-tumor mechanism of *TCB* flavonoids.

At present, more than 200 chemical components have been isolated from TCB. The research has shown that TCB contains a rich variety of chemical substances, which form the basis for TCB to exhibit its medicinal effects. The chemical components in TCB are mainly flavonoids, organic acids, alkaloids, coumarins, sterols, phenylethanes, polysaccharides, and ceramides. The main chemical components of TCB are shown in Table 3, Table 4, Table 5 and Table 6 and Figure 3, Figure 4, Figure 5, Figure 6 and Figure 7.

### 2.3. Flavonoids

TCB is rich in flavonoids. Many of these flavonoids have a glycoside structure along with flavanols, dihydroflavonoids, and flavonoid oxygen glycosides [35,36,37]. These compounds are formed by combining the sugar moiety and flavonoid moiety at the C-8 position through a C-C glycosidic bond. According to the oxygenation degree of the central three-carbon chain, the location of the B-ring connection (second or third position), and whether the three-carbon chain is looped or not, flavonoids are classified into 16 types, including flavonoids, flavonols, dihydroflavonoids, dihydroflavonols, chalcones and dihydrochalcones, isoflavones, and anthocyanins [38]. About 100 types of flavonoid components have been identified from TCB (Figure 3, Supplementary Table S1), including orientin, *Bauhinia* glycoside, hypericum glycoside, lignocerotin, quercetin, apigenin, and rhamnetin [39]. Orientin accounts for ~10%, and oysterin accounts for ~2% of total flavonoids in *Citrus aurantium* [40].

### 2.4. Organic Acids

#### 2.4.1. Phenolic Acids

Phenolic acids are principally derivatives of benzoic acid and can be divided into two categories. The first category lacks a free hydroxyl group, including veratric acid, benzoic acid, and veratric acid methyl ester (methyl veratrate). The second category includes a free hydroxyl group, including vanillic acid and methyl-*p*-hydroxy-benzoate (Figure 3; Table 4) [41,42,43]. Researchers have used high-speed countercurrent chromatography to separate resveratrol and vanillic acid from TCB [44], and there are also studies using high-performance countercurrent chromatography to separate caffeoylquinic acids [45]. Wang isolated a new compound from TCB: chrysanthemum glucoside [46]. Li Danyi also isolated benzoic acid by silica gel column chromatography [47]. Wei separated methyl veratelic acid, methyl protocatechuic acid p-hydroxybenzoic acid, and p-hydroxybenzoic acid by silica gel column chromatography and preparative thin-layer chromatography [9].

**Table 3:** Information relating to phenolic acid compounds in TCB.

No.	Compound Name	Nucleus	Supersede	References
1	Veratric acid	XXIX	R1=COOH; R2=OCH_3_; R3=OCH_3_; R4=H	[42]
2	Methyl veratric acid	XXIX	R1=COOCH_3_; R2=OCH_3_; R3=OCH_3_; R4=H	[42]
3	Vanillic acid	XXIX	R1=COOH; R2=OCH_3_; R3=OH; R4=H	[42]
4	Protocatechuic acid	XXIX	R1=COOH; R2=OH; R3=OH; R4=H	[42]
5	Gallic acid	XXIX	R1=COOH; R2=OH; R3=OH; R4=OH	[47]
6	Benzoic acid C_6_H_5_COOH	XXIX	R1=COOH; R2=H; R3=H; R4=H	[42]
7	4-Hydroxybenzoic acid	XXIX	R1=COOH; R2=H; R3=OH; R4=H	[47]
8	Methyl para-hydroxybenzoate	XXIX	R1=COOCH_3_; R2=H; R3=OH; R4=H	[47]
9	Methyl 3,4-dihydroxybenzoate	XXIX	R1=COOCH_3_; R2=OH; R3=OH; R4=H	[47]
10	4-Hydroxy-2,6-dimethoxybenzaldehyde	XXIX	R1=CHO; R2=OCH_3_; R3=OH; R4=OCH_3_	[47]
11	Aureate acid	XXXI		[48]
12	Eleutheroic acid	XXX	R1=OH; R2=OCH_3_	[48]
13	Amaryllis glycoside	XXX	R1=O-β-D-glucopyranosyl; R2=OCH_3_	[48]
14	4-(β-D-Glucopyranoside)-3-(3-methyl-2-butenyl) benzoic acid	XXX	R1=O-β-D-glucopyranosyl; R2=H	[42]
15	Ursolic acid	XXXIII		[47]
16	Anisic acid	XLII		[47]
17	Vitamin C	XLI		[47]
18	(2R,3S)-Piscidicacid	XXXIX		[47]
19	3-(6-hydroxy-7- methoxy-2H-1,3- benzodioxol-5-yl) propanoicacid	XXXVIII		[47]
20	Isochlorogenic acid A	XXXV		[47]
21	Veratric acid glucose ester	XXXII		[49]
22	Iso rosmarinic acid glycoside	XXXVII		[47]
23	Crystal orchid glycosides	XL		[47]
24	Hookerine	XXXVI		[47]
25	Florissim (name)	XXXIV		[47]

#### 2.4.2. Fatty Acids

In one study, the GC-MS method was used to separate and identify fatty acids from a 95% ethanol extract of TCB, in which the content of saturated fatty acids that was isolated accounted for 57.95% of the detected substances, and unsaturated fatty acids accounted for 30.3% [49]. In the research of Peng, dextrose veratricate was also isolated from TCB (Figure 5; Table 5) [50]. In further research, Wang used gas chromatography–mass spectrometry (GC-MS) to separate and characterize the fatty acid components in TCB. A total of 31 fatty acid components were identified in the 95% ethanol extract of TCB, among which 21 saturated fatty acid components accounted for 57.95%, with significant quantities of cetanoic acid (palmitic acid) and tetradecanoic acid [49]. There were nine unsaturated fatty acids that provided a total content of 30.35%, namely, oleic acid, linoleic acid, and palmitoleic acid (Figure 5; Table 4).

**Table 4:** Information relating to fatty acid compounds in TCB.

No.	Compound Name	Nucleus	Supersede	References
1	Methyl caprylate	XLIX	R=H	[49]
2	Methyl decanoate	XLIX	R=CH_2_CH_3_	[49]
3	Methyl dodecanoate	XLIX	R=(CH_2_)_3_CH_3_	[49]
4	Methyl tridecanoate	XLIX	R=(CH_2_)_4_CH_3_	[49]
5	Methyl tetradecanoate	XLIX	R=(CH_2_)_5_CH_3_	[49]
6	Methyl pentadecanoate	XLIX	R=(CH_2_)_6_CH_3_	[49]
7	Methyl hexadecanoate	XLIX	R=(CH_2_)_7_CH_3_	[49]
8	Methyl heptadecanoate	XLIX	R=(CH_2_)_8_CH_3_	[49]
9	Methyl octadecanoate (Methylstearate)	XLIX	R=(CH_2_)_9_CH_3_	[49]
10	Methyl eicosanoate (Methylarachidonate)	XLIX	R=(CH_2_)_11_CH_3_	[49]
11	Methyl docosanoate	XLIX	R=(CH_2_)_13_CH_3_	[49]
12	Methyl tetracosanoate	XLIX	R=(CH_2_)_15_CH_3_	[49]
13	Methyl benzoate	XLVII	R1=H, R2=H	[49]
14	Methyl 3,4-dimethoxybenzoate	XLVII	R1=OCH_3_, R2=OCH_3_	[49]
15	Methyl 3-phenyl-2-propenoate	LI	R1=H, R2=H	[49]
16	Methyl 3-(4-hydroxyphenyl)-2-propenoate	LI	R1=H, R2=OH	[49]
17	Methyl (4-hydroxy-3- methoxyphenyl)-2-propenoate	LI	R1=OCH_3_, R2=OH	[49]
18	Methyl 2-hydroxyhexadecanoate	LV	R1=OH, R2=H, R3=H	[49]
19	Methyl 3-hydroxyhexadecanoate	LV	R1=H, R2=OH, R3=H	[49]
20	Methyl 10-hydroxyhexadecanoate (Methyl palmitate)	LV	R1=H, R2=H, R3=OH	[49]
21	(Z)-9-Hexadecenoic acid methyl ester (methyl palmitoleate)	LVI	R=(CH_2_)_4_CH_3_	[49]
22	(Z)-9-Octadecenoic acid methyl ester (Methyl oleate)	LVI	R=(CH_2_)_6_CH_3_	[49]
23	(Z, Z)-9,12-Octadecadienoic acid methyl ester (methyl linoleate)	LVI	R=CH=CH(CH_2_)_4_CH_3_	[49]
24	(Z, Z, Z)-9,12,15- Octadecatrienoic acid methyl ester	LVI	R=CH=CHCH_2_CH=CHCH_2_CH_3_	[49]
25	Hexadecanoic acid	LIX	R1=H, R2=H	[49]
26	(R)-10,16- Dihydroxyhexadecanoic acid	LIX	R1=OH, R2=OH	[49]
27	Dimethyl succinate	XLIII		[49]
28	Dimethyl octanedioate	XLIV		[49]
29	Dimethyl azelate	XLV		[49]
30	Dimethyl 3-hydroxy-2-methylglutarate	XLVI		[49]
31	Methyl phenylacetate	XLVIII		[49]
32	2,3-Dihydrobenzofuran	L		[49]
33	Methyl 4-phenyl-2-butenoate	LII		[49]
34	9-(O-Propylphenyl)-nonanoic acid methyl ester	LX		[49]
35	(E)-11-Eicosenoic acid methyl ester	LVIII		[49]
36	Methyl 2-methoxy-eicosatrizoate	LXI		[49]
37	(Z, Z, Z)-9,12,15-Octadecatrien-1-ol	LVII		[49]
38	2-Hydroxybenzaldehyde oxime	LIV		[49]
39	4-Hydroxyacetophenone	LIII		[49]

### 2.5. Indole Alkaloids

TCB alkaloids are a type of isoquinoline alkaloid component first discovered from TCB, which have significant antiviral and antibacterial activities [51]. TCB alkaloids have strong antioxidant and antibacterial activities [52]. In addition, (R)-Cyanomethyl-3-hydroxyoxindole [2], isoquinoline alkaloids, such as aurantiamine (trolline) [51], adenine, and other substances have been identified (Table 5) [53].

**Table 5:** Information relating to alkaloids in TCB.

No.	Compound Name	Nucleus	References	Structures
1	Adenine	LXIV	[53]	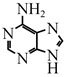
2	(R)-Cyanomethyl-3-hydroxyoxindole	LXII	[2]	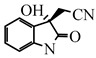
3	Clenbuterol	LXIII	[1]	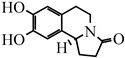

### 2.6. Other

The majority of studies examining the activities of TCB’s constituents have focused on flavonoid and phenolic acids. There are fewer studies that have reported on the polysaccharides within TCB. In addition, TCB also contains coumarin, residual alcohols, terpenoids, phenols, ceramides, and polysaccharides. Jia [54] investigated the extraction method, isolation, purification, in vitro antioxidant activity, and related pharmacological activities of polysaccharides obtained from TCB. The coumarin compounds in TCB include chicory glycosides, hesperidin, sterols, 6-deoxy-D-manno-1, 4-lactone, 6-deoxy-D-manno-1,4-lactone, lactone, tetracosane, and trolliamide [55]. Wang [50] isolated and identified a pure polysaccharide from TCB using GC-MS. The monosaccharides of the pure polysaccharide were rhamnose, D-galactose, and arabinose (Figure 6; Table 6).

**Table 6:** Information relating to the other compounds in TCB.

No.	Compound Name	Nucleus	Supersede	References
1	3,4-Dihydroxyphenethyl alcohol	LXVIII	R = H	[56]
2	2-(3,4-Dihydroxyphenyl) ethanol glucoside	LXVIII	R = Glc	[53]
3	4-Hydroxy-3-methoxyphenethyl alcohol	LXIX		[53]
4	2-(3,4-Dihydroxyphenyl)-ethyl-O-beta-D-glucopyranose	LXX		[42]
5	3,5-Dihydroxyphenethylalcohol 3-O-β-D-glucopyranoside	LXXI		[42,57]
6	4′-O-(6″-O-vanillyl-β-D-glucopyranosyl) phenethyl alcohol	LXXII		[42]
7	Trolliamide	LXXIII		[26]
8	Trolliusol A	LXXIV		[26]
9	Heptaphyllum lactone	LXV		[9]
10	β-Sitosterol	LXVI		[36]
11	Carotene	LXVII		[36]
12	Rhamnose	LXXV		[58]
13	D-Galactose	LXXVI		[58]
14	Arabinose (type of sugar)	LXXVII		[58]
15	Vanillin	LXXVIII		[59]

## 3. Pharmacology

### 3.1. Antimicrobial Effects

In addition, researchers have employed in vitro experimental methods to evaluate the inhibitory effects of either the extracts or the compounds of TCB on bacteria and fungi. Liu et al. conducted a comparative study of the in vitro antimicrobial and antioxidant activity of the total flavonoids of TCB and its main flavonoid constituents (orientin and vitexin). They concluded that the total flavonoids of orientin and vitexin showed significant inhibitory effects against Gram-positive bacteria and exhibited high antioxidant activity. Overall, the bactericidal potency and antioxidant activity of orientin were better than that of vitexin, which may, therefore, be the main component that elicits its purgative and detoxifying properties [60]. The aqueous 60% ethanol and 95% ethanol extracts of TCB showed different inhibitory effects on Gram-positive and Gram-negative bacteria. The inhibitory effect of water and 60% ethanol extracts on Gram-positive bacteria was significantly greater than that on Gram-negative bacteria, and the minimum inhibitory concentration (MIC) of the water extract on *Staphylococcus aureus* was 3.9 mg/L [61].

There is scope for researchers to also explore the antimicrobial mechanism of action of TCB through observation of its effects on microbial cell membranes, enzyme systems, or gene expression. The MICs of total flavonoids of *S. aureus*, *Bauhinia* glycoside, and orientin on *S. aureus* and *Staphylococcus epidermidis* were, in mg/L, 50 and 25, 100 and 25, and 25 and 25, respectively. Poncirin and orientin have both shown activity against *Pseudomonas aeruginosa*, *Escherichia coli*, and *Shigella dysenteriae* [62]. The emergence and spread of drug-resistant bacteria has become a global problem. Several studies have already explored the potential antimicrobial activity of plant species against drug-resistant bacteria, such as methicillin-resistant *Staphylococcus aureus*. The total flavonoids of TCB have strong effects in vitro against *S. epidermidis*, *S. aureus*, *S. dysenteriae*, and group-B streptococci, which are common clinical infections. In vivo, the total flavonoids of TCB can inhibit *S. aureus* infections and significantly reduce the mortality rate of mice at 48 h [63]. Auriculine has obvious effects on Gram-positive and Gram-negative bacteria, and its MICs for *S. aureus*, *Klebsiella pneumoniae*, and *Streptococcus pneumoniae* have been reported to be 32, 128, and 128 mg/L, respectively [64]. The proglobeflowery acids veratridine and auriculine have shown moderate effects on *S. aureus* and *S. epidermidis* [62].

### 3.2. Antiviral Effects

Several studies have isolated and characterized the extracts or active ingredients of TCB to identify specific compounds with antiviral activity. The effects of antiviral substances on viral replication, host cytokine production, and immune response have also been investigated, which may lead to the development of antiviral drugs.

TCB is often used in the treatment of infections of the upper respiratory tract, namely, pharyngitis, and tonsillitis. The proprietary medicines of TCB, Jinlianhua granules/capsules, are also commonly used in a clinical setting. However, the mechanism of action against infections of the upper respiratory tract and influenza has not been elucidated. Network pharmacology and molecular docking have been utilized to explore the pharmacological basis and potential targets of the anti-influenza effect of *Acer japonicum*. In the constructed drug–component–target–disease network, β-sitosterol, liubiquinol, phytocannabinoid acid, apigenin, and quercetin were found to have obvious roles. These data indicate that these five components might be the main material basis of the efficacy of *A. japonicum*. Yang [65] found that β-sitosterol had a significant inhibitory effect on the inflammatory response mediated by the influenza A (H1N1) virus. Experimental research undertaken with animals showed that β-sitosterol significantly inhibited the lung injury and inflammatory response induced by the influenza A (H1N1) virus. Quercetin, apigenin, and willow-through-fish xanthophylls are flavonoids. Several studies have shown that natural flavonoids have significant antiviral effects. Liu [66] found that apigenin and lignans have strong inhibitory effects against the influenza virus (H3N2). Apigenin can also inhibit the replication of the hepatitis-C virus via the downregulation of the expression of mature microRNA122 and has no significant effect on cell growth [67]. An aqueous extract of TCB showed a significant inhibitory effect on Coxsackie B virus (CoxB3), with a half-maximal inhibitory concentration (IC_50_) of 0.318 mg/mL. At a concentration of 0.061–1.953 mg/mL, the antiviral effect upon CoxB3 was positively correlated with the concentration. There was no antiviral effect at <0.031 mg/mL. There was also a significant inhibitory effect on a variant of CoxA24 (Coxsackie virus A24) [68]. An alcohol extract of TCB had a direct killing effect on the PR8 strain of influenza virus A in vitro at a concentration of 25–200 g/L and inhibited the proliferation of this strain in chicken embryos at a concentration of 25–400 g/L [69]. Another investigation showed that a 60% ethanol extract and total flavonoids of *C. aurantium* had weak antiviral activity against parainfluenza virus type 3, with IC_50_ values of 77.5 and 74.6 μg/mL, and selectivity index (SI) values of 2.0 and 1.0, respectively [70]. Orientin and vitexin showed strong activity against activity on parainfluenza type 3, with IC_50_ values of 11.7 and 20.8 μg/mL and SI values of 32.1 and 16.0, respectively. 2″-O-(2-methyl butyryl)iso-neoxanthin, chrysin, and proto-Trollius acid showed inhibitory effects against influenza-A viruses, with IC_50_ and SI values of 74.3 μg/mL and 7.17 [57], 56.8 μg/mL and 4.81 [51], and 184.2 μg/mL and 4.0, respectively [50].

### 3.3. Anti-Tumor Effects

Researchers have sought to reveal the molecular mechanisms by which TCB exerts its anti-tumor activity. Additionally, the effects of TCB are being explored on cancer-cell apoptosis, cell-cycle regulation, and angiogenesis to understand its anti-tumor mechanism. There are also studies investigating the application of TCB with other anti-tumor drugs/treatments.

TCB flavonoids have shown significant inhibitory effects on the proliferation of tumor cells (K562, HeLa, Ec-109, and NCI-H446) cultured in vitro in a dose-dependent manner in the concentration range from 0.793 to 12.688 g/L. TCB flavonoids can cause an increase in the number of K562 cells in the G0/G1 phase and a decrease in those in the S phase. The cycle of K562 cells can be blocked in the G0/G1 phase, thus inhibiting their proliferation [71]. TCB flavonoids can also dose-dependently inhibit the growth of A549 cells and upregulate the expression of the oncogene p53, downregulating the expression of the oncogene B cell lymphoma-2 (Bcl-2), and inducing the apoptosis of A549 cells [72]. TCB flavonoids have obvious inhibitory effects on human breast cancer (MCF-7) cells, with three main mechanisms of action (Figure 7). First, TCB flavonoids can downregulate the protein expression of nuclear factor-kappa B (NF-κB) and Bcl-2 and increase the protein expression of caspase-3 and caspase-9 to induce apoptosis [73]. Second, TCB flavonoids can inhibit the proliferation and promote the apoptosis of MCF-7 cells through the inhibition of the poly(ADP-ribose) polymerase-1/p53 signaling pathway [74]. Third, TCB flavonoids can inhibit the activity of telomerase reverse transcriptase (TERT) and reduce telomerase activity, thus inhibiting the proliferation and inducing the apoptosis of MCF-7 cells [75]. Orientin and poncirin show positive effects against the growth of esophageal cancer (EC-109) cells and can induce their apoptosis [76]. The yellow pigment in TCB can block the synthesis of nitrosamine and can scavenge free radicals, which have anti-cancer effects [77].

### 3.4. Antioxidant Effects

Research has shown that the phenolic acid and flavonoid components in the 80% ethanol extract of TCB have a strong free radical scavenging ability. The median effective concentration of these components for superoxide anion, hydroxide radical, lipid free radical R, and single oxygen is 46.00 mg/mL, 5.64 mg/mL, 5.19 mg/mL, and 3.97 mg/mL, respectively. Therefore, the components’ ability to scavenge these free radicals is significantly better than that of the antioxidant vitamin C. The effective concentration of this extract for DPPH is 44 mg/mL, which is also significantly better than that of the antioxidant butylhydroxytoluene (EC: 52 mg/mL) [78]. The flavonoids in TCB have shown obvious antioxidant effects on lard, and the flavonoid dose is positively correlated with antioxidant activity. If the concentration of flavonoids is 0.5%, then the antioxidant effect is in accordance with the antioxidant effect of butylated hydroxytoluene (BHT). Flavonoids have a synergistic effect with both vitamin C (VC) and BHT [79]. Researchers have conducted in-depth tests on the antioxidant properties of various extracts of TCB, including a 70% ethanol extract, a 70% ethanol ultrasonic extract, a 70% ethanol microwave extract, and a water extract. The TCB 70% ethanol ultrasound extract was found to have the strongest antioxidant capacity. Furthermore, detailed measurements were taken of the content of total flavonoids, total polyphenols, quercetin, quercetin 2-0-galactoside, and hyperoside in the TCB extract. The antioxidant capacity of TCB was determined to be significantly positively correlated with the content of total flavonoids, total polyphenols, and these four identified flavonoid monomers [80].

### 3.5. Anti-Inflammatory and Analgesic Effects

TCB can reduce the production of pro-inflammatory mediators, reduce the inflammatory response, and may have an adjunctive therapeutic effect on some inflammation-related diseases (e.g., arthritis, dermatitis). Some substances in TCB can inhibit the release of pro-inflammatory mediators, regulate the inflammation-related signaling pathways, and reduce tissue damage. Some studies have discovered that TCB may have a regulatory effect on the immune system and inflammation-related cytokines; therefore, it has the capacity to reduce the degree of the inflammatory response.

A decoction of TCB and the pharmacodynamic components of TCB (orientin-2″-O-β-L-galactoside, orientin, glycosides, other flavonoids, and other organic acid compounds) have anti-inflammatory and analgesic effects. These components have been shown to reduce xylene-induced ear swelling in mice and egg-white-induced toe swelling, demonstrate strong phagocytosis of mononuclear macrophages in mice, and inhibit leukocyte phagocytosis in the vesicles of mice; however, they have weak effects on central analgesia. A different decoction of TCB and the pharmacodynamic components of TCB also alleviated the pain caused by acetic acid in mice; however, it again showed a weaker effect on central analgesia. The mechanism of action may involve the inhibition of the early inflammation caused by capillary hyperpermeability, exudation, and edema and may also inhibit the inflammation caused by leukocytes and the enhanced phagocytosis of mononuclear macrophages [81]. Ouabain, orientin, veratric acid, and TCB glycosides have inhibitory effects on the ear swellings caused by croton oil in mice, and the order of anti-inflammatory activity is ouabain > TCB glycosides > veratric acid > orientin [82]. In addition, high doses of aqueous and 70% ethanol extracts of the stems and leaves of TCB were shown to inhibit ear swellings in mice [83]. 2″-O-(2‴-methylbutyryl) iso-Japanese swertiamarin, 2″-O-(2‴-methylbutyryl)-iso-adamanthin, and 2″-O-(3‴,4‴-dimethoxybenzoyl) oysteroside (10 mg/kg) inhibited the ear swelling induced with TPA (phorbol ester) in mice by 58.6%, 35.5%, and 27.6%, respectively, and these three compounds also showed anti-inflammatory effects [84].

### 3.6. Antipyretic Effect

The total flavonoids of TCB significantly reduced the body temperature of febrile rabbits induced with *E. coli* endotoxins in a dose-dependent manner. A postulated mechanism of action could be the inhibition of the production/release of tumor necrosis factor-α and interleukin-1β induced by *E. coli* endotoxins, followed by the inhibition of the production/release of prostaglandin-E2. These actions would lower the abnormally elevated thermoregulatory point, decrease heat production, increase heat dissipation, and restore the body temperature to normal [85]. The pharmacology of TCB is summarized in Table 7.

## 4. Traditional Applications

In its traditional application, TCB is good at clearing heat and removing toxins and can be used for the treatment of carbuncle sores, sore throats, mouth sores, eye redness, and other diseases. In the Zhao Bing Nan formula for nourishing Yin and lowering fire, the existence of TCB is described as a Chinese herbal medicine. For example, for swelling and pain in the eyes, golden lotus flowers and wild chrysanthemums can be mashed and applied to the eye sockets [98]. In 2003, the State Administration of Traditional Chinese Medicine announced a prescription for the prevention of atypical pneumonia. This TCB “soup” consisted of Wrightia laevis (10 g), *Pueraria mirifica* (10 g), Perilla leaf (6 g), *Taraxacum mongolicum* (15 g), and TCB (6 g), which can clear heat, remove toxins, and disperse wind [7].

The classic Tibetan book *Jingzhu Materia Medica* recorded that TCB “heals sores, stops pulse heat, treats abscesses, clears heat from the upper body, treats head injuries, and nourishes the meridians” [99]. TCB is also used by Mongolian, Tibetan, Miao, Tujia, Korean, Uyghur, Kazakh, Oroqen, Lisu, and other ethnic groups under a variety of names and effects (Table 8).

## 5. Clinical Applications

According to Pharmaceutical Intelligence (https://www.yaozh.com/, accessed on 31 January 2024), medicines in China containing TCB include chewable tablets, capsules (hard and soft), lozenges, oral liquids, dispersible tablets, drops, and granules. Proprietary Chinese medicine prescriptions also include Ulan Thirteen-flavored Tangsan, Shuangjinlian Combination, TCB Heat-Clearing Effervescent Tablets, TCB Heat-Clearing Granules (standard and sugar-free), and TCB Punch. A comparison of proprietary Chinese medicines of TCB included in the 2020 edition of the *Chinese Pharmacopoeia* [13] is presented in Table 9. TCB can be used to treat upper respiratory tract infections, wind and heat colds, mumps, oral ulcers, etc.

### 5.1. Upper Respiratory Tract Infections

Studies have shown that both TCB granules and Jinlianhua tablets can significantly shorten the duration of the clinical symptoms of fever, sore throats, nasal congestion, and coughs in the treatment of upper respiratory tract infections [96,103]. TCB granules combined with cefuroxime sodium can also be used in the treatment of children for treating acute upper respiratory tract infections and reducing toxicity at the same time, thus reducing the adverse drug reactions caused by antibiotics [104].

### 5.2. Wind and Heat Colds

In treating wind and heat colds, TCB tablets and capsules are significantly effective in relieving the specific symptoms of sore throats, congestion in the pharyngeal mucosa, and tonsil enlargement [105] and as a safe and effective general medicine. A relevant study showed that a total of 827 patients were included in five studies. The meta-analysis showed that there was no heterogeneity among the studies, and the Jinlian flower was superior to the control group in improving the clinical symptoms of influenza wind-heat, with fewer adverse reactions [106].

### 5.3. Mumps

The research has identified that the combination of TCB granules and Ribavirin injections can quickly alleviate childhood mumps. The clinical symptoms were improved, and the time to disappearance of parotid swelling and fever was shortened without any significant adverse reactions, resulting in significant clinical efficacy [107].

### 5.4. Treatment of Oral Ulcers

The total effective rate of TCB capsules combined with dexamethasone acetate tablets in the treatment of recurrent oral ulcers is as high as 94.7%, and the recurrence rate of patients after 6 months is about 75% lower than that of patients treated with dexamethasone acetate tablets alone [108]. In the study of Jianli Zhang and her team [109], it was discovered that the combination of TCB capsules and allicin capsules had a total effective rate of 93% in the treatment of recurrent oral ulcers, and the duration of oral ulcers was effectively shortened together with a reduction in patients’ pain.

## 6. Safety Evaluation

In recent years, the unique curative effects of TCM formulations have garnered increasing attention, followed by interest in their quality control and safety. The testing of acute toxicity (mice injected with 80 times the human dose for 48 h) did not elicit any adverse reactions. The testing of subacute toxicity (rabbits injected with 20 times the human dose for 20 days) did not elicit significant effects on levels of gamma-glutamyltransferase, non-protein nitrogen in serum, or hematology parameters, thereby indicating that the toxicity of TCB was very low [110].

There are studies indicating that the extract of TCB has good antibacterial and antibiofilm activity against Streptococcus mutans, and its 30% alcohol extract shows the best effect at the minimum dose without toxic effects [88]. In the subacute toxicity study of TCB on rats, combined with biochemical indicators and general observations, it is known that TCB has no toxic effects [111]. In addition, TCB and antibiotic treatment of upper respiratory tract infections in children safety studies showed that the drug safety is high, suitable for clinical widely used [104].

## 7. Quality Control

At present, with the research of drug effect, the management of medicinal materials has been paid attention to. Therefore, the research methods of TCB effective composition and quality management is especially critical. We summarize the fingerprint and chemical composition determination and analysis methods. These are the methods of TCB identification and provide a theoretical basis for subsequent quality standards of TCB.

### 7.1. Determination of Flavonoid Content

The mass fraction of flavonoids in TCB can be as high as 15%. The highest flavonoid content is attributable to orientin and *Bauhinia* glycoside [112], which have antioxidant, bacteriostatic, and anti-tumor activities [92,94,113]. These flavonoids can be efficiently absorbed in the intestine [114]. Therefore, these two flavonoids are used as the index components in the quality evaluation of TCB.

In order to evaluate the indicator components’ content in TCB, most methods are based on high-performance liquid chromatography (HPLC). HPLC has the advantages of good reproducibility, high stability, and a high degree of automation, and the use of the process facilitates standardization. In the 2020 edition of the Chinese Pharmacopoeia, HPLC is extensively mentioned. In most studies, the number of detected components is one–five. However, the qualitative and quantitative detection methods of TCB using HPLC established by Song [43] can simultaneously detect 19 flavonoid components, which significantly improves the comprehensive nature of the quality evaluation.

### 7.2. Determination of Volatile Components

Volatile components not only have an aromatic odor but also have various pharmacological effects, which is one of the important indicators for evaluating floral herbs. Ji [115] discovered that 38 volatile components, including lauric acid, tetradecanoic acid, hexadecenoic acid ethyl ester, and eicosanoids, were found in *A. auricula*. Qin [116] established a quantitative model of the relationship between structure and chromatographic retention for these 38 volatile components using the molecular valence connectivity index and atomic type electrical topological state index. Hou [117] used GC-MS to analyze differences in the type and content of fatty acids between TCB produced in Liaoning and Inner Mongolia in China. They found that TCB produced in Liaoning contained 11 fatty acids (75.08% of the total detected amount), and TCB produced in the Inner Mongolia Autonomous Region contained 13 fatty acids (72.41% of the total detected amount). The two differential fatty acids were pentadecanoic acid and acetylenic acid, suggesting that the volatile components of *A. auricula* were significantly affected by their habitat.

### 7.3. Determination of Micronutrient Content

TCB is not only used for medicinal purposes but also as a tea, in which the trace elements are water-soluble components. Trace elements are closely related to the normal metabolism of the human body and play an important role in the prevention and treatment of diseases. The types and contents of trace elements are the result of the genetic factors of organisms, the abundance of elements in habitats, and the need for selective absorption during the growth stage of species. Therefore, it is rational to evaluate the quality of TCM formulations from the perspective of trace elements [118].

Li [119] found that TCB contains eight essential elements (Fe, Mg, Cu, Zn, Mn, Cr, Pb, and As) and that the content of various essential trace elements in TCB is much higher than that identified in grains, vegetables, and meat. Liang [118] and colleagues compared the contents of K, Ca, Mg, Fe, Zn, Mn, Cu, and other elements in samples of artificially planted and wildflowers of the *Trollius chinensis* species and found that they were identical. These data indicate that, based on the level of inorganic elements, artificially planted products can meet the requirements for both medicinal use and teas. Li [120] found that the contents of Ca and Fe in TCB from different origins differed slightly; meanwhile, the differences in the contents of Mn, Cu, and Zn were more significant.

### 7.4. Chemical Fingerprint

The fingerprinting of TCM formulations can reflect their chemical components. This strategy avoids the limitation of evaluating the quality of herbs by an individual or one type of component and is widely recognized by the industry. The fingerprinting of TCB is based mainly on the use of HPLC. Compared with HPLC, ultra-performance liquid chromatography (UPLC) has the advantages of faster detection (detection time can be reduced by >30%) and higher efficiency. Lei [121] carried out a fingerprinting study of the TCB species using UPLC. Thirty-eight batches of plants (including TCB, *Trollius ledebourii*, and *Trollius altaicus*) were studied, and twenty-six characteristic peaks were observed. The peak with the largest area was taken as the reference peak. Combined with the chemometrics of clustering analysis, it was possible to rapidly identify CB, *Trollius ledebourii*, and *Trollius altaicus*. Yuan [122] combined the efficient separation ability of HPLC with the chemical composition identification of MS to establish the HPLC fingerprint of TCB. They observed 32 common peaks and realized the qualitative and quantitative analyses of 13 components.

## 8. Conclusions and Future Perspectives

Due to the scarcity and destruction of wild resources of TCB species, the medicinal herbs of TCB species on the market are quite varied. Pharmacological research has been suggested as necessary for the TCB species to find alternative sources and expand the source of the medicinal herbs of *Trollius chinensis* species. Artificial cultivation can also be carried out to meet the market demand. However, due to the small seeds and low-temperature dormancy characteristics of the species, seedling emergence is difficult. In-depth research on seedling technology is needed to carry out large-scale artificial cultivation. Some studies have shown that the total flavonoid content of the stems and leaves of the artificially cultivated *Trollius chinensis* species and the short-valved *Trollius chinensis* species in Beijing reached their maximum value in the second half of June at 6.34% and 5.82%, respectively. Therefore, the pharmacological activity of the stems and leaves harvested in the second half of June can be investigated further, which can be used as a new source of the *Trollius chinensis* species to alleviate the pressure on the market.

In addition to the wide use of TCB in clinical practice, *T. asiaticus*, *T. macropetalus*, *T. farreri*, *T. ledebouri*, *T. buddae*, and *T. ranunculoides* are also used as medicines. Some studies [123] have shown that flavonoids and alkaloids are found in all varieties of *T. altaicus*, but organic acids have been found only in *T. altaicus*, *T. buddae*, and *T. altaicus*; meanwhile, coumarins and phenylethanes have been reported only in *T. altaicus* and *T. buddae*. The reports on pharmacological activity have focused mainly on *T. altaicus* and *T. shortifolia*. The pharmacological effects of TCB have centered mostly on their flavonoid components. There has been less frequent research on the pharmacological effects of other active ingredients (polysaccharides and coumarins) in TCB. The anti-inflammatory, analgesic, antioxidant, antibacterial, antiviral, anti-tumor, and antipyretic effects of TCB components merit further investigation.

This study provides a review of the studies covering the herbal nature, phytochemistry, pharmacology, traditional uses, clinical application, and quality control of TCB, intending to lay a theoretical foundation for the development of new clinical applications for TCB.

## Figures and Tables

**Table 1:** TCB in the 1977 edition of Chinese Pharmacopoeia and local Drug Standards.

Protozoa	Exual Flavors, Attributes, and Functions	References
TCB	Bitter, slightly cold. Antibacterial and anti-inflammatory. Used for upper respiratory tract infection, pharyngitis, tonsils stomatitis, otitis media, acute conjunctivitis, and acute lymphangitis.	Chinese Pharmacopoeia (1977) [13]
TCB	Bitter, slightly cold. Clears heat and removes toxins. Used for upper respiratory tract infection, pharyngitis, tonsil stomatitis, otitis media, acute conjunctivitis, and acute lymphangitis.	Shanxi Standard of Traditional Chinese Medicinal Materials (1987) [14]
TCB	Bitter, slightly cold. Attributed to the liver meridian. Clears heat and disperses wind, detoxifies and subdues swellings, calms the liver, and improves eyesight. For mouth sores and swollen throats, floating heat, and tooth pronouncement. Earache and eye pain, mountain miasma, boils, fire poisoning, upper respiratory tract infections, acute enteritis, urinary infections sores, abscesses, etc.	Beijing Standard of Traditional Chinese Medicine [15]
TCB	Bitter, slightly cold. Clears heat and removes toxins. Used for upper respiratory tract infections, tonsillitis, pharyngitis, otitis media, mouth sores, and boils.	Jiangsu Standard of Traditional Chinese Medicine [16]
TCB	Bitter, cold. Attributed to the liver meridian. Clears heat and disperses wind. Detoxifies and subdues swelling, calms the liver, and improves eyesight. Used for mouth sores, swollen throats, floating fever, toothache, earache, eye pain, mountain miasma, boils, and fire.	Processing Specification of Chinese Herbal Decoction Pieces in Sichuan Province (2002) [17]
TCB	Bitter, cold. Attributed to lung and stomach meridians. Clears heat. Detoxification. Indications: Wind and heat colds, throat swelling and pain, mouth sores, eye redness, and swelling.	Zhejiang Chinese Medicine Preparation (2015) [18]
TCB	Bitter, slightly cold. Attributed to the lung and stomach meridians. Clears heat and detoxifies. For upper respiratory tract infections. Pharyngitis, tonsillitis, otitis media, etc., as well as mouth sores and boils.	Ningxia Standard of Traditional Chinese Medicine (2017) [19]
TCB	Bitter, slightly cold. Attributed to the lung, liver, and gallbladder meridians. Clears heat and removes toxins. Used for lung-heat cough, sore throat, eye redness and swelling, and pus in ears.	Hubei Province Traditional Chinese Medicine Quality Standard (2018) [20]
TCB	Bitter, slightly cold. Attributed to the lung and stomach meridians. Clears heat and detoxifies. For sore throat, eye redness, swelling and pain, phlegm-heat tuberculosis, scrofula and mouth sores, and swelling and pain in the ear.	Shanghai Processing Standard of Chinese Herbal Decoction Pieces (2018) [21]
TCB	Bitter, slightly cold. Attributed to lung and stomach meridians. Antibacterial and anti-inflammatory. For upper respiratory tract infections, pharyngitis, tonsillitis, otitis media, acute conjunctivitis, and acute lymphadenitis.	Processing Standard of Traditional Chinese Medicine Decoction Pieces in Anhui province (2019) [22]
*Trollius asiaticus* L. *Trollius macropetalus Fr.* *Schmidt. Trollius ledebourii Reichb.*	Bitter, slightly cold. Clears heat and removes toxins. Used for carbuncles, swollen sores, sore throat, mouth sores, and redness of the eyes.	Standard of Traditional Chinese Medicine in Heilongjiang Province (2001) [23]

**Table 2:** Distribution of plants of the genus TCB.

**No.**	**Genus**	**Latin Name**	**D** **istribution Area**
1	Saussurea involucrata	*Trollius chinensis* Bunge	Shanxi, northern Henan, Hebei, eastern Nei Mongol, western Liaoning, and Jilin, China
2	Wide-petaled TCB	*Trollius asiaticus* L.	Heilongjiang, Xinjiang, etc., China; Mongolia; Siberia; and Russia
3	Amaryllis longifolia	*Trollius macropetalus Fr.* *Schmidt*	Liaoning, Jilin, and Heilongjiang, China
4	Short-petaled TCB	*Trollius ledebouri Reichb.e*	Heilongjiang and northeastern Inner Mongolia, China; eastern Siberia; the Far East; and Russia
5	Garden nasturtium	*Trollius farreri Stapf*	Northwestern Yunnan (Wisi and Deqin), western Sichuan, northeastern Tibet, southern and eastern Qinghai, southern Gansu, and southern Shaanxi (Qinling), China
6	Altaic TCB	*Trollius altaicus* L.	Northern Xinjiang and western Inner Mongolia, China
7	Sichuan and Shaanxi TCB	*Trollius buddae Schipcz*	Northern Sichuan, southern Gansu, and southern Shaanxi, China
8	Dsungaripus communis	*Trollius dschungaricus* *Regel*	Tianshan, Zhaosu, and Xinjiang, China; Central Asia; and Russia
9	Tropaeolum majus	*Trollius japonicus Miq*	Changbai Mountain and Jilin, China; Sakhalin Island, Japan
10	Small-flowered TCB	*Trollius micranthus Hand. Mazz*	NW Yunnan and E Tibet, China
11	Little TCB	*Trollius pumilus D. Don*	Southern Tibet, China
12	Ranunculus	*Trollius ranunculoides Hemsl*	Northwestern Yunnan, eastern Tibet, western Sichuan, southern and eastern Qinghai, and southern Gansu, China
13	Taiwan TCB	*Trollius taihasenzanensis* *Masamune*	China–Taiwan
14	TCB	*Trollius vaginatus*	NW Yunnan (Zhongdian) and SW Sichuan (Muli), China
15	Yunnan TCB	*Trollius yunnanensis* *(Franch.) Ulbr.*	Western and northwestern Yunnan and western Sichuan, China
16	Long-petaled Yunnan TCB	*Trollius yunnanensis var. eupetalus*	Gongshan, Deqin, and NW Yunnan, China
17	Yunnan TCB (var.)	*Trollius yunnanensis (Franch.) Ulbr. var. anemonifolius (Brühl) W.T. Wang*	Western Sichuan and southern Gansu, China
18	Peltate-leaved Yunnan TCB (var.)	*Trollius yunnanensis (Franch.) Ulbr. var. peltatus W. T. Wang*	Emei and Sichuan, China
19	Qinghai–Tibetan TCB (var.)	*Trollius pumilus var. tanguticus Bruhl*	Northeastern Tibet, northwestern Sichuan, southern and eastern Qinghai, and southwestern Gansu, China
20	Amaryllis (var.)	*Trollius pumilus D. Don var. foliosus (W. T. Wang) W. T. Wang*	Min County and Southern Gansu, China
21	Derg TCB (var.)	*Trollius pumilus D. Don var. tehkehensis (W. T.Wang) W. T. Wang*	Alpine grassland around Dege and Sichuan, China
22	Dwarf goldenrod (var.) with large leaves	*Trollius farreri Stapf var. major W. T. Wang*	NW Yunnan (Deqin) and SE Tibet (Tsatsumi), China
23	P11111ale purple gilded lily (Amaryllis lilaca)	*Trollius lilacinus* Bunge	Tian Shan and Xinjiang, China; Western Siberia, Russia; and Central Asia
24	Water lily	*Nymphoides aurantiacum* *(Dalz. Ex Hook.) O. Kuntze*	China, Taiwan, western India, and Sri Lanka
25	European Golden Lotus (Hemerocallis fulva)	*Trollius europaeus*	Northern, central, and western Europe
26	Amaryllis	*Trollius laxus*	Distributed within the U.S. in Connecticut, Del. (Delaware), NJ (New Jersey), N.Y (New York), Pa. (Pennsylvania), and Ohio

**Table 7:** The pharmacological action of TCB.

Pharmacological Effects	Extracts/Compounds	Types	Animal/Cell	Dosage	Effects	Reference
Antimicrobial effects	Crude extract	In vivo	BALB/mice	0.2 mg/g	TCB crude extract exerted anti-influenza virus effect by regulating the expression of TLR3, IRF3, IFN-β, TAK1, and TBK1 in the TLR3 signaling pathway.	[86]
	Trochinenol A	In vitro	293 T cells	1.0 mg	Trochinenol A has a minimum inhibitory concentration (MIC) of 6.25 mg/mL against Staphylococcus aureus and can inhibit the TNFα-induced NF-κB pathway.	[87]
	Crude extract	In vitro	Bacterium suspension	3.125 mg/mL	TCB extracts of streptococcus mutans have antibacterial activity and resistance to biofilm activity.	[88]
	Flavonoids	In vitro	Mice	440 mg·kg^−1^	An inhibitory effect on Staphylococcus aureus infection was shown.	[63]
Antiviral effects	Crude extract	In vivo	Mice	0.2 mg/g	TCB crude extract exerted anti-influenza virus effect by regulating the expression of TLR3, IRF3, IFN-β, TAK1, and TBK1 in the TLR3 signaling pathway.	[4]
	Crude extract	In vitro	MDCK cells	0.5 mg·mL^−1^	Reduced the expression of tlr3 mRNA and protein, as well as IFN β mRNA, by intervening in the TLR3 signaling pathway.	[89]
	Veratric acid; vitexin; trolline	In vivo	SD mice	400 μmol/L	Downregulating tlr3 and tlr7 signaling pathways and upregulating TLR4 signaling pathway exerted anti-H1N1 virus effect.	[90]
Anti-tumor effects	Flavonoids	In vivo	MCF-7 breast cancer cells	0.0991, 0.1982, 0.3964, 0.7928 or 1.5856 mg/mL	Flavonoids selectively reduced the expression of bcl-2 and NF-κB while increasing the expression of caspase-9 and caspase-3.	[91]
	Orientin, vitexin	In vitro	EC-109 cells	5.0, 8.0 µM	Orientin and vitexin inhibited the growth of EC-109 cells and induced early apoptosis.	[92]
	Flavonoids	In vitro	MCF-7 cells	100, 200, 300 μg·mL^−1^	Inhibition of PARP-1/p53 pathway inhibited the proliferation of human breast cancer MCF-7 cells.	[74]
	Orientin	In vitro	EC-10 cells	5, 10, 20, 40, 80 μmol/L	Orientin dose-dependently inhibited the growth and proliferation of EC-109 cells and induced apoptosis of tumor cells.	[93]
Antioxidant effects	Orientin, vitexin	In vitro	Mice	40 mg/kg	40 mg/kg dose of prince’s-feather and vitex glycosides has the same antioxidant capacity and vitamin e.	[78]
	Flavonoids	In vitro	Mice	44 μmol/L	TCB total flavonoids showed strong ROS scavenging activity.	[94]
	Orientin, vitexin	In vivo	Mice	200 mg/kg^−1^	Significantly increased the serum T-AOC activity of D-galactose-induced senescence mice.	[95]
Antipyretic effects	Orientin, vitexin	In vivo	Mice	200 mg·kg^−1^	Significantly increased the serum T-AOC activity of D-galactose-induced senescence mice.	[96]
	Formononetin	In vitro	NSCLC cells	0.5, 1.5, 3, 10 μg/mL	Inhibition of EGFR-Akt-Mcl-1 axis to suppress tumor growth in non-small cell lung cancer.	[97]
Anti-inflammatory and analgesic effects	Flavonoids	In vitro	RAW264. 7 cells	62.5, 125, 250, 500, 1000 μg/mL	Both total flavonoids and total phenolic acids of TCB had obvious anti-inflammatory activities, and total flavonoids were slightly better than total phenolic acids.	[46]

**Table 8:** A list of medicinal uses of TCB by ethnic groups.

Ethnic Group	Pharmaceutical Name	Botanical Name	Efficacy	Reference
Tibetan	Laibganzipomedo, capital of Malawi (Tw)	Saussurea involucrata	It heals sores, stops pulse heat, cures abscesses, and clears heat from the upper half of the body.	Jingzhu Materia Medica [100]
	Maddox Sitchin, Maddox, clear as a bell	Garden nasturtium	Flowers for food poisoning, sores, carbuncles, wounds, and ulcers. The whole herb for pus and swelling.	Dictionary of Chinese Ethnic Medicines
	Coloration of the charts	Amaryllis Grandiflora	Flowers and fruits for gallbladder pain and meat poisoning.	Dictionary of Chinese Ethnic Medicines
	Pungent color, Meadow color Khin, Medoselkyn, Murtoseljian	Ranunculus	Flowers for food poisoning, sores and carbuncles, traumatic ulcers, yellow water disease, various toxic diseases, infectious diseases, fever, and biliousness. Eradicates botulism, black aconite poison and poisonous fever.	National Chinese Herbal Medicine List Dictionary of Chinese Ethnic Medicines [101]
	Sekchin, Medoselqing	Little T.chinensis	The flowers cure food poisoning, sores and carbuncles, and traumatic ulcers.	Dictionary of Chinese Ethnic Medicines
	The coloration of the charts	Yunnan TCB	The flowers or fruits are used for food poisoning, febrile illnesses, bilious fever, and cholecystitis.	Chinese Materia Medica
Mongolian medicine	Alatanhua Khiqiqi, Modgas Rizhin, Xiezhi Khiqiqi	Saussurea involucrata	Flowers for gold wounds, traumatic infections, bloody eyes, throat fever, sore throats, otitis media, acute lymphadenitis, acute conjunctivitis, feverish toothache, and sores.	Chinese Materia Medica—Mongolian Medicine Volume [30]
	Pungent color, Meadow color Khin, Medoselkyn, Murtoseljian	Ranunculus	Flowers for food poisoning, sores and carbuncles, traumatic ulcers, yellow water disease, various toxic diseases, infectious diseases, fever, and biliousness. Eradicates botulism, black aconite poison, and poisonous fever.	Chinese Materia Medica [101] National Chinese Herbal Medicine List Dictionary of Chinese Ethnic Medicines
	Mado Sekchin, Medoselqing	Little TCB	The flowers cure food poisoning, sores and carbuncles, and traumatic ulcers.	Dictionary of Chinese Ethnic Medicines
	The coloration of the charts	Yunnan TCB	The flowers or fruits are used for food poisoning, febrile illnesses, bilious fever, and cholecystitis.	Chinese Materia Medica
Mongolian medicine	Alatanhua Khiqiqi, Alatanhua Khiqiqi, Modgas Rizhin, Xiezhi Khiqiqi	Saussurea involucrata	Flowers for gold wounds, traumatic infections, bloody eyes, throat fever, sore throats, otitis media, acute lymphadenitis, acute conjunctivitis, feverish toothache, and sores.	Chinese Materia Medica—Mongolian Medicine Volume [101]
The Koreans	Broad-petal gold plum	Wide petaled TCB	Whole herbs and flowers for epilepsy and conjunctivitis.	Dictionary of Chinese Ethnic Medicines
	Gold flower	Short-petaled TCB	Whole herbs and flowers for epilepsy, hemorrhages, and conjunctivitis.	Dictionary of Chinese Ethnic Medicines
Lisu medicine	Yunnan mother cilia, chicken claw grass	Yunnan TCB	Root for malaria; whole plant for external wind-cold, rheumatism and numbness, and cervical lymph nodes.	Dictionary of Chinese Ethnic Medicines
	Mother (name) Seeley	Ranunculus	Tuberculosis. Roots, flowers, and fruits for meat poisoning and malaria. The whole plant treats rheumatism and lymph node tuberculosis; the stem, leaves, and flowers treat rheumatism and numbness, lymph node tuberculosis and chicken-claw wind; the flowers are powdered to treat suppurative wounds.	Dictionary of Chinese Ethnic Medicines
Oroqen or Orochon (ethnic group)	Kahoot, Kimmelweed, Gold Bumps	Short-petaled TCB	Flowers for sore throats, mouth sores, eye redness, carbuncle sores, bronchitis, cholecystitis, chronic tonsillitis, acute otitis media, acute conjunctivitis, and acute lymphadenitis.	Dictionary of Chinese Ethnic Medicines
Kazakh ethnic group of Xinjiang	Saussurea involucrata	Altaic TCB	Flowers are used for acute and chronic tonsillitis, pharyngitis, upper respiratory tract infections, otitis media, conjunctivitis, lymphadenitis, fever reduction, acute bronchitis, acute appendicitis, acute enteritis, urinary tract infections, sores, and carbuncles.	Dictionary of Chinese Ethnic Medicines
Uighur (Uyghur) ethnic group of Xinjiang	Xiehedan (name)	Altaic TCB	The flowers are used for fever, pharyngitis, tonsillitis, acute bronchitis, otitis media, conjunctivitis, acute appendicitis, acute enteritis, urinary tract infection, sores, and carbuncles.	Dictionary of Chinese Ethnic Medicines
Tujia ethnic group of Hunan	Inborn son	Saussurea involucrata	Flowers for upper respiratory tract infections, tonsillitis, pharyngitis, acute otitis media, chronic tympanitis, acute lymphadenitis, acute conjunctivitis, mouth sores, and boils.	Jingzhu Medica [100] Dictionary of Chinese Ethnic Medicines [102]
Hmong or Miao ethnic group of southwest China	Aravana-qiqiqi, Drynaria, and Golden Plumweed	Saussurea involucrata	Flowers treat acute and chronic tonsillitis, acute otitis media, acute periostitis, acute conjunctivitis, acute lymphadenitis, upper respiratory tract infections, tonsillitis, and pharyngitis.	Jingzhu Materia Medica [100] Dictionary of Chinese Ethnic Medicines [102]

**Table 9:** TCB-related proprietary Chinese medicine prescriptions.

Name	Functions and Indications	Formula	Method of Making	Content Determination Standard
TCB Fever Granules	Clear heat and remove toxins, promote the production of fluids, and relieve phlegm. Used for colds and flu with high fever, thirst, dry throat, sore throat, cough, and thick phlegm and influenza and upper respiratory tract infections with the above symptoms.	TCB 600 g, Big Green Leaf 600 g, Gypsum 450 g, Zhi Mu 300 g, Di Huang 300 g, Xuan Shen 300 g, Fried Bitter Almond 450 g.	Aqueous decoction with dextrin and meringue to make granules.	Each 1 g contains no less than 0.32 mg of Bauhinia glycosides.
TCB Lozenges	Clear heat and remove toxins, subdue swelling and relieve pain, and reduce sore throats. It is used for the redness, swelling, and pain of the pharynx, the swelling of gums, and sores of mouth and tongue caused by internal heat and toxin; acute pharyngitis, acute tonsillitis, and upper respiratory tract infections with the above symptoms.	TCB 450 g, Peppermint Oil 4 mL	Aqueous decoction with menthol oil and magnesium stearate pressed tablets.	Orientin is not less than 1.6 mg per tablet.
TCB Oral Liquid	Clears heat and removes toxins. Used for upper respiratory tract infections, pharyngitis, and tonsillitis caused by wind or heat and toxicity attacking the lungs and internalized heat and toxicity.	TCB 450 g	Add honey or simple syrup to the decoction, and sodium benzoate, ethyl hydroxybenzene, and water.	Orientin is not less than 1.5 mg per 1 mL.
TCB Granules	Ibid	TCB 1000 g	Water decoction with sucrose and dextrin to make granules.	Orientin is not less than 16.0 mg per sachet.
TCB Capsules	Ibid	TCB 1000 g	Aqueous decoction with dextrin in capsules.	Orientin is not be less than 3.0 mg per capsule.
TCB Flakes	Ibid	TCB 1500 g	Decoction of water with starch to make granules and then pressed tablets.	Orientin is not less than 4.5 mg per tablet.

## Data Availability

No data were used for the research described in the article.

## Supplementary Materials

The following supporting information can be downloaded at: https://www.mdpi.com/article/10.3390/ph17060800/s1, Table S1: The structure of flavonoids in TCB.
