# Supplementary material for: A Comprehensive Research Review of Herbal Textual Research, Phytochemistry, Pharmacology, Traditional Uses, Clinical Application, Safety Evaluation, and Quality Control of Trollius chinensis Bunge
Source: Pharmaceuticals (Basel). 2024 Jun 18;17(6):800. doi: 10.3390/ph17060800 (PMC11206471; doi:10.3390/ph17060800)
Supplement: Supplementary file 1 [file pharmaceuticals-17-00800-s001.zip › pharmaceuticals-3044864-supplementary.pdf]

No	Compound Name	Nucleus	Supersede	References
1	Orientin-2″-O-b eta-L-galactoside	I	R1=OH; R2=OH; R3=L-gal; R4=H; R5=H; R6=OH	[43-45]
2	Orientin-2″-O-b eta-D-xylopyrano side	I	R1=OH; R2=OH; R3=D-xyl; R4=H; R5=H; R6=OH	[44]
3	Orientin-2″-O-beta-D-glucopyran oside	I	R1=OH; R2=OH; R3=D-glu; R4=H; R5=H; R6=OH	[44]
4	Orientin-2″-O-β-arabinopyrano side	I	R1=OH; R2=OH; R3=D-ara; R4=H; R5=H; R6=OH	[44]
5	Orientin-6″-O-glucoside	I	R1=OH; R2=OH; R3=L-gal; R4=H; R5=H; R6=OH	[45]
6	Orientin	I	R1=OH; R2=OH; R3=H; R4=H; R5=H; R6=OH	[45-47]
7	2″-O-Acetyl Ori entin	I	R1=OH; R2=OH; R3=Ac; R4=H; R5=H; R6=OH	[46]
8	3″-O-Acetyl Ori entin	I	R1=OH; R2=OH; R3=H; R4=Ac; R5=H; R6=OH	[46, 48]
9	6″-O-Acetyl Ori entin	I	R1=OH; R2=OH; R3=H; R4=H; R5=Ac; R6=OH	[39, 46, 48]
10	Bauhinia-2″-O-beta-L-galacto side	I	R1=OH; R2=H; R3=L-gal; R4=H; R5=H; R6= OH	[45, 46]
11	Oryzanin-2″-O-beta-D-xylopyrano side	I	R1=OH; R2=H; R3=D-xyl; R4=H; R5=H; R6= OH	[45, 46]
12	Vitexin-2″-O-beta-D-glucopyrano side	I	R1=OH; R2=H; R3=D-glu; R4=H; R5=H; R6= OH	[43]
13	Oryzanin-2″-O- β-arabinopyrano side	I	R1=OH; R2=H; R3=D-ara; R4=H; R5=H; R6= OH	[44]
14	Vitexin-6″-O-glucoside	I	R1=OH; R2=H; R3=H; R4=H; R5=D-glu; R6=OH	[46, 48]
15	Bauhinia pseudoa cacia	I	R1=OH; R2=H; R3=H; R4=H; R5=H; R6=OH	[39, 49]
16	2″-O-Acetylpodo phyllotoxin	I	R1=OH; R2=H; R3=Ac; R4=H; R5=H; R6=OH	[45, 46]
17	3″-O-Acetylpodophyllotoxin	I	R1=OH; R2=H; R3=H; R4=Ac; R5=H; R6=OH	[45, 46]
18	6″-O-Acetylpodo phyllotoxin	I	R1=OH; R2=H; R3=H; R4=H; R5=Ac; R6=OH	[46, 48]
19	Isodangenoxin (a nti-inflammatory drug used in treating asthma)	I	R1=OCH3; R2=H; R3=H; R4=H; R5=H; R6=OH	[39, 45, 46, 50]
20	Iso-Nippon Danga nese	I	R1=OCH3; R2=OH; R3=H; R4=H; R5=H; R6=OH	[45, 46]
21	Acacia-7-O-beta- D-galactopyranos ide	I	R1=H; R2=H; R3=H; R4=H; R5=H; R6=OCH_3	[39]
22	TrollisinI	I	R1=OCH3; R2=H; R3=H; R4=H; R5=H; R6=OH	[51]
23	3″-O-(2‴-methylbutanoyl)orienti n	II	R1=OH; R2=OH	[46, 48]
24	3″-O-(2‴-methylbutanoyl) ouabain	II	R1=OH; R2=H	[2, 43]
25	3″-O-(2‴-methyl butanoyl) isodang enoxanthin	II	R1=OCH_3; R2=H	[2, 43, 52]
26	3″-O-(2‴-methyl butyryl)iso-niho nbenzoin	II	R1=OCH_3; R2=OH	[51]
27	2″-O-Feruloyl Orientin	III	R1=OH; R2=OH; R3=OCH_3	[44]
28	2″-O-Feruloylpo dophyllotoxin	III	R1=H; R2=OH; R2=OCH_3	[44]
29	2″-O-(3‴-Methox ycaffeoyl) ouabain	III	R1=H; R2=OH; R3=OH	[39]
30	2″-O-Feruloyl isodanaxanthin	III	R1=H; R2=CH_3O; R3=OCH_3	[44]
31	2″-O-Feruloylis ojaponicotin	III	R1=OH; R2=CH_3O; R3=OCH_3	[39]
32	2″-O-(2‴-methyl butanoyl) orientin	IV	R1=OH; R2=OH; R3=OH; R4=CH_2CH_3	[45-47]
33	4′-Methoxy-2″- O-(2‴-methylbutanoyl) orientin	IV	R1=OH; R2=OH; R3=OCH3; R4=CH_2CH_3	[45]

34	2″-O-(2‴-methyl butanoyl)ouabain	IV	R1=OH; R2=H; R3=OH; R4=CH_2CH_3	[45]
35	4′-Methoxy-2″-O(2‴methylbutanoyl) ouabain	IV	R1=OH; R2=H; R3=OCH_3; R4=CH_2CH_3	[48]
36	2″-O-(2‴-methyl butanoyl)isodang anin	IV	R1=OCH_3; R2=H; R3=OH; R4=CH_2CH_3	[48]
37	2″-O-(2‴-methyl butanoyl)iso-nih onobenzoin	IV	R1=OCH_3; R2=OH; R3=OH; R4=CH_2CH_3	[45]
38	2″-O-Isopropylb enzoyl isodanaxa nthin	IV	R1=OCH_3; R2=OH; R3=OH; R4=CH_3	[45]
39	2″-O-(3‴,4‴-dimethoxybenzoyl) or ientin	V	R1=OH; R2=OH; R3=OH; R4=OCH_3	[48]
40	2″-O-(3‴,4‴-Dimethoxybenzoyl) ponaxoside	V	R1=OH; R2=H; R3=OH; R4=OCH_3	[53]
41	2″-O-(3‴,4‴-Dim ethoxybenzoyl) is odangenoxanthin	V	R1=OCH_3; R2=H; R3=OH; R4=OCH_3	[45]
42	2″-O-(3‴,4‴-dim ethoxybenzoyl)is o-nihonbenzoin	V	R1=OCH_3; R2=OH; R3=OH; R4=OCH_3	[45]
43	2″-O-vanillyl o rientin	V	R1=OH; R2=OH; R3=OH; R4=OH	[45]
44	2″-O-Vanilloylp seudolariciresinol	V	R1=OH; R2=H; R3=OH; R4=OH	[45]
45	2″-O-Vanilloyli sodomorhodopsin	V	R1=OCH_3; R2=H; R3=OH; R4=OH	[53]
46	apigenin	VI	R1=H; R2=OH; R3=H; R4=OH; R5=H; R6=H; R7=OH; R8=H	[44]
47	Robinia pseudoac acia	VI	R1=H; R2=OH; R3=H; R4=OH; R5=H; R6=H; R7=OCH_3; R8=H	[45, 53]
48	Coelenterazine (loanword)	VI	R1=H; R2=OH; R3=H; R4=OH; R5=H; R6=OCH_3; R7=OH; R8=H	[54]
49	Sophorin 7-O-neo hesperidin	VI	R1=H;R2=O-neohesperidoside;R3=H; R4=OH; R5=H; R6=OH; R7=OCH_3;R8=H	[45]
50	Sophorin 7-O-rutinoside	VI	R1=H;R2=Orutinoside;R3=H;R4=OH;R5=H;R6 =OH; R7=OCH_3; R8=H	[45]
51	8-C-β-D-Xylopyr anose acuminatin	VI	R1=D-xyl; R2=H; R3=H; R4=OH; R5=H; R6=OH; R7=OCH_3; R8=H	[44]
52	Salvia divinorum	VI	R1=H; R2=OCH_3; R3=OCH_3; R4=OH; R5=H; R6=H; R7=OCH_3; R8=H	[44, 54]
53	thistle flavoring	VI	R1=H; R2=OCH3; R3=OCH_3; R4=OH; R5=H; R 6=H; R7=OH; R8=H	[54]
54	rhinocerosin	VI	R1=H; R2=OH; R3=H; R4=OH; R5=H; R6=OH;R7=OH; R8=H	[44]
55	Lycopene	VI	R1=H; R2=OH; R3=OCH_3; R4=OH; R5=H; R6= H; R7=OCH_3; R8=H	[44]
56	Xindi Omin	VI	R1=H; R2=O-rutinoside; R3=H; R4=OH; R5=H; R6=H; R7=OCH_3; R8=H	[44]
57	quercetin (phosp holipid found in quercetin)	VI	R1=H; R2=OH; R3=H; R4=OH; R5=OH; R6=OH; R7=OH; R8=H	[44]
58	naringenin	VI	R1=H; R2=OH; R3=H; R4=OH; R5=H; R6=H; R7=OH; R8=H	[54]
59	kaempferol	VI	R1=H; R2=OH; R3=H; R4=OH; R5=OH; R6=H; R7=OH; R8=H	[54]
60	lycopene	VI	R1=H; R2=OH; R3=H; R4=OH; R5=OH; R6=OH; R7=OH; R8=OH	[54]
61	Farnisin	VI	R1=H; R2=OH; R3=H; R4=H; R5=H; R6=OH; R7=OCH_3; R8=H	[54]
62	geranylgeranyl	VI	R1=H; R2=OH; R3=H; R4=OH; R5=H; R6=OH; R7=OCH_3; R8=H	[54]
63	Quercetin-3-O-be ta-D-glucopyrano side	VI	R1=H; R2=OH; R3=H; R4=OH; R5=O-β-D-glucopyrano-side; R6=OH; R7=OH; R8=H	[44]

64	Quercetin-3-O-beta-L-rhamno side	VI	R1=H; R2=OH; R3=H; R4=OH; R5=O-β-L-rhamnoside; R6=OH; R7=OH; R8=H	[44]
65	5-Hydroxy-4′,7,8-trimethoxyflav one	VI	R1=OCH_3; R2=OCH3; R3=H; R4=OH; R5=H; R 6=H; R7=OCH_3; R8=H	[54]
66	4′,5-Dihydroxy- 7,8-dimethoxyfla vone	VI	R1=OCH3; R2=OCH3; R3=H; R4=OH; R5=H; R 6=H; R7=OH; R8=H	[54]
67	hypericin	VII	R1=OH; R2=OH; R3=CH_2OH	[48]
68	Echinacea purpur ea	VII	R1=OH; R2=H; R3=CH_2OH	[48]
69	Ka empfe rol 3 -(6″-ethylglucur onide)	VII	R1=OH; R2=H; R3=$COOCH_2CH_3$	[48]
70	TrochiyosideA	VIII	R1=OH; R2=OH; R3=H; R4=OH	[48]
71	TrochiyosideB	VIII	R1=OH; R2=OCH_3; R3=OCH_3; R4=OH	[45]
72	TrochiyosideC	VIII	R1=OH; R2=H; R3=OCH_3; R4=OCH_3	[45, 55]
73	2″-O-(6‴-O-caffeoyl)-galactopranosylvitexin	VIII	R1=H; R2=OH; R3=H; R4=OH	[55]
74	2″-O-(6‴-O-feru loyl)-galactopy anosylorientin	VIII	R1=OH; R2=OCH_3; R3=H; R4=OH	[55]
75	Acacia-7-O-beta-D-glucoside	IX	R1=OH; R2=OCH_3	[53]
76	Soyoside	IX	R1=H; R2=OH	[55]
77	soybean lutein	X	R1=OH; R2=OCH_3; R3=H; R4=H	[55]
78	soy isoflavone	X	R1=OH; R2=H; R3=H; R4=H	[55]
79	4′,5-Dihydroxy- 3′,7-dimethoxyi soflavone	X	R1=OCH_3; R2=H; R3=OH; R4=OCH_3	[55]
80	3″-O-veratroylv itexin	XI	R=H	[54]
81	3″-O-veratroylo rientin	XI	R=OH	[54]
82	6‴-(3-Hydroxy-3- ethylglutaryl)- 2″-O-beta-D-gal actopyranosyl orientin	XII.	R=OH	[44]
83	6‴-(3-Hydroxy-3- ethylglutaryl)- 2″-O-β D-galac topyranosylpseudomonas glycoside	XII	R=H	[40, 55]
84	Isohomoflavin-2″-O-(6--ferul oyl)-β-L-galact oside	XIII		[53]
85	2″-O-veratroylv itexin	XIV		[53]
86	3″-O-Feruloylpodophyllotoxin	XV		[53]
87	2″-O-Benzoylisojaponicotin	XVI		[53]
88	2″-O-(6‴-O-veratroyl)-galactopy ranosylvitexin	XVII		[53]
89	2″-O-(2‴-O-meth ybutyryl)-glucop yranosyl isoswer tisin	XVIII		[53]
90	(2E)-2-methyl-1-O-vanilloyl-4-β-D-glucopyrano-side-2-butene	XIX		[53]
91	kumquat lucoside	X		[53]
92	Apigenin-8- C –(2″-O-feruloyl)-β-D-glucoside	XI		[53]
93	Orientin-2″-O-beta-L-galactopyr anoside	XII		[53]
94	(-)-Gallocatechi	XIII		[44]
95	Lycopodium album	XIV		[44]
96	Isomartyrin	XV		[44]
97	Neocarlinoside	XVI		[44]
98	6″-Malonylcosmo siin	XVII		[44]
99	Glucosylorientin	XVIII		[44]
